# Functional Annotation, Genome Organization and Phylogeny of the Grapevine (*Vitis vinifera*) Terpene Synthase Gene Family Based on Genome Assembly, FLcDNA Cloning, and Enzyme Assays

**DOI:** 10.1186/1471-2229-10-226

**Published:** 2010-10-21

**Authors:** Diane M Martin, Sébastien Aubourg, Marina B Schouwey, Laurent Daviet, Michel Schalk, Omid Toub, Steven T Lund, Jörg Bohlmann

**Affiliations:** 1 Michael Smith Laboratories, University of British Columbia, 2185 East Mall, Vancouver, B.C, V6T 1Z4, Canada; 2 Wine Research Centre, University of British Columbia, 2205 East Mall, Vancouver, B.C., V6T 1Z4, Canada; 3 Unité de Recherche en Génomique Végétale (URGV) UMR INRA 1165 - Université d'Evry Val d'Essonne - ERL CNRS 8196, 2 Rue Gaston Crémieux, 91057 Evry Cedex, France; 4 Firmenich SA, Corporate R&D Division, Geneva, CH-1211, Switzerland

## Abstract

**Background:**

Terpenoids are among the most important constituents of grape flavour and wine bouquet, and serve as useful metabolite markers in viticulture and enology. Based on the initial 8-fold sequencing of a nearly homozygous Pinot noir inbred line, 89 putative terpenoid synthase genes (*VvTPS*) were predicted by *in silico *analysis of the grapevine (*Vitis vinifera*) genome assembly [1]. The finding of this very large *VvTPS *family, combined with the importance of terpenoid metabolism for the organoleptic properties of grapevine berries and finished wines, prompted a detailed examination of this gene family at the genomic level as well as an investigation into *VvTPS *biochemical functions.

**Results:**

We present findings from the analysis of the up-dated 12-fold sequencing and assembly of the grapevine genome that place the number of predicted *VvTPS *genes at 69 putatively functional *VvTPS*, 20 partial *VvTPS*, and 63 *VvTPS *probable pseudogenes. Gene discovery and annotation included information about gene architecture and chromosomal location. A dense cluster of 45 *VvTPS *is localized on chromosome 18. Extensive FLcDNA cloning, gene synthesis, and protein expression enabled functional characterization of 39 *VvTPS*; this is the largest number of functionally characterized *TPS *for any species reported to date. Of these enzymes, 23 have unique functions and/or phylogenetic locations within the plant *TPS *gene family. Phylogenetic analyses of the *TPS *gene family showed that while most *VvTPS *form species-specific gene clusters, there are several examples of gene orthology with *TPS *of other plant species, representing perhaps more ancient *VvTPS*, which have maintained functions independent of speciation.

**Conclusions:**

The highly expanded *VvTPS *gene family underpins the prominence of terpenoid metabolism in grapevine. We provide a detailed experimental functional annotation of 39 members of this important gene family in grapevine and comprehensive information about gene structure and phylogeny for the entire currently known *VvTPS *gene family.

## Background

Terpenoids are a large class of metabolites that are involved in the fragrance and aroma constituents of flowers and fruits, plant defense, and primary plant metabolism [2-4]. Although all terpenoids arise from a few structurally simple prenyldiphosphate precursors, an enormous assortment of thousands of possible molecules comes to fruition. This chemical diversity of terpenoid structures is attributed, in large part, to the myriad ways of folding and the eventual quenching of reactive carbocation intermediates in the reaction catalyzed by terpenoid synthases (TPS) [5,6]. The products of TPS can be further modified by other enzymes such a cytochrome P450 dependent monooxygenases and various transferases.

The initial 8-fold sequencing and assembly of a grapevine (*Vitis vinifera *L.) inbred Pinot noir genome (PN40024) lead to the prediction of 89 grapevine *TPS *(*VvTPS*) genes, which mirrors a vibrant role for terpenoid secondary metabolism in grapevine biology [7,8]. For example, in wine made from aromatic grape varieties, monoterpene alcohols such as linalool, geraniol, and cis-rose oxide impart important floral flavour qualities [9]. Sesquiterpenes have also been identified as important indicators of grape aroma. Recently, Parker *et al. *[10] identified α-ylangene as a sesquiterpene metabolite marker associated with peppery aroma and taste in Australian Shiraz grape berries, and the sesquiterpene ketone, rotundone, was found to be the compound responsible for this attribute in peppery/spicy Australian Shiraz grapes and wines [11]. Monoterpenes such as 1,8-cineol, and sesquiterpenes such as α-humulene, β-caryophyllene as wells as α- and γ-muurolene have been described in Cabernet Sauvignon pre-veraison berries [12]. Similar profiles of terpene volatiles have also been documented from the headspace above Chardonnay leaves, flowers, and green berries [13]. The presence of some terpenes early in berry development and in other parts of the plant may indicate a role in defense. Terpenoid volatiles are released from grapevines following insect feeding [14] or the application of methyl jasmonate [15].

Of all plant species for which genome sequences are available, the *TPS *gene family has been comprehensively explored only in *Arabidopsis thaliana*, in which 32 intact *AtTPS *genes were identified [16]; functions have been established for several of these genes [17-20]. A number of *TPS *genes have also been characterized against the background of the sequenced rice (*Oryza sativa*) genome [21-26] which has at least 40 *TPS*-like sequences identified (S. Aubourg unpublished results). For comparison, genome sequence analysis of poplar (*Populus trichocarpa*) identified 47 *TPS *genes [27], only two of which have been functionally characterized [28,29]. Prior to the sequencing of a grapevine genome, we reported on the cDNA cloning and product profiles of three *VvTPS *[30,31] and we detailed the involvement of valencene synthase, a sesquiterpene synthase, in the evolution of grapevine floral scent [32], but a comprehensive analysis of *VvTPS *has not been reported thus far.

The importance of terpenoids in grapevine biology and wine flavour and quality motivated a genome-wide inventory and functional characterization of the *VvTPS *gene family. We present the manually curated annotation of the *VvTPS *gene family from the current 12-fold genome sequence coverage. This work defines 69 putatively functional *VvTPS*, 20 partial *VvTPS*, and 63 probable *VvTPS *pseudogenes including *VvTPS *gene architecture and chromosome localizations. The *VvTPS *gene family shows extensive gene duplication and in many instances, functional diversification across all subfamilies except those involved in primary metabolism (subfamilies *TPS-c *and *TPS-e*). Conclusions regarding diversification are supported by phylogenetic analyses of the *VvTPS *family and functional characterization of heterologously expressed VvTPS proteins.

## Results and Discussion

### Genome-wide identification of *TPS *genes in *Vitis vinifera*

Screening of the predicted proteome and the six-frames-translated 12-fold genome sequence of *V. vinifera *with protein sequences of previously characterized TPS identified 152 loci exhibiting significant similarities with known TPS (see the Methods section for details). Our annotation of the 152 *TPS*-like gene models (Additional File 1) classified them into four types: (i) 53 are complete *VvTPS *genes that contain the expected functional motifs and domains [16,33,34] required to render them functional; (ii) 16 are complete *VvTPS *genes but the ORFs contain a frameshift or premature stop codon either due to a point mutation or a possible sequencing error; (iii) 20 are partial *TPS *genes disrupted by sequence gaps or located in scaffold extremities; and (iv) 63 are obvious pseudogenes disrupted by numerous deletions, frameshifts and/or stop codons (Additional File 1). After removing the genes of this last type, the number of potentially functional *VvTPS *ranges from a minimum of 53 up to 89 genes. The missing sequences of the partial genes (group iii) prevented meaningful sequence alignments and gene classification; therefore, we removed them from our further analysis which focused on the 69 *VvTPS *genes of groups (i) and (ii). The presence of cognate EST and/or cDNA sequences provides proof of transcription for 40 (58%) of them (Additional File 1).

The relatively high gene sequence and structure conservation across the plant *TPS *family [16] allow us to be confident in the result of the genome-wide *VvTPS *gene prediction, combining automatic and manual annotations. Manual curation and evaluation have substantially improved the identification of *VvTPS *genes: For example, out of the 69 *VvTPS *genes 12 were missed by the automated pipeline used for annotation of the grapevine genome [1,35]. Furthermore, intron-exon structures of 40 *VvTPS *genes required manual correction to obtain complete and consistent coding sequences. The results of the *VvTPS *gene annotations confirmed a large *VvTPS *gene family previously predicted from the 8-fold genome assembly [1] and expand the previous estimation of the *VvTPS *family size. While a lower estimate of only 35 *VvTPS *genes was reported from the analysis of a second heterozygous Pinot noir genome sequence [36], the sequence information available for this genome in NCBI GenBank http://www.ncbi.nlm.nih.gov/genbank also revealed about 70 *VvTPS*. A comparison with other plant genomes in which *TPS *genes have been annotated showed that the grapevine *VvTPS *gene family is the largest identified to date. The *Arabidopsis thaliana *genome contains 32 complete *AtTPS *genes and eight *AtTPS *pseudogenes [16], while the rice and poplar genomes are predicted to encode 40 to 50 *TPS*-like genes according to [27] and unpublished results (S. Aubourg).

### Annotation of *VvTPS *relative to the overall plant *TPS *gene family

**Figure 1:**
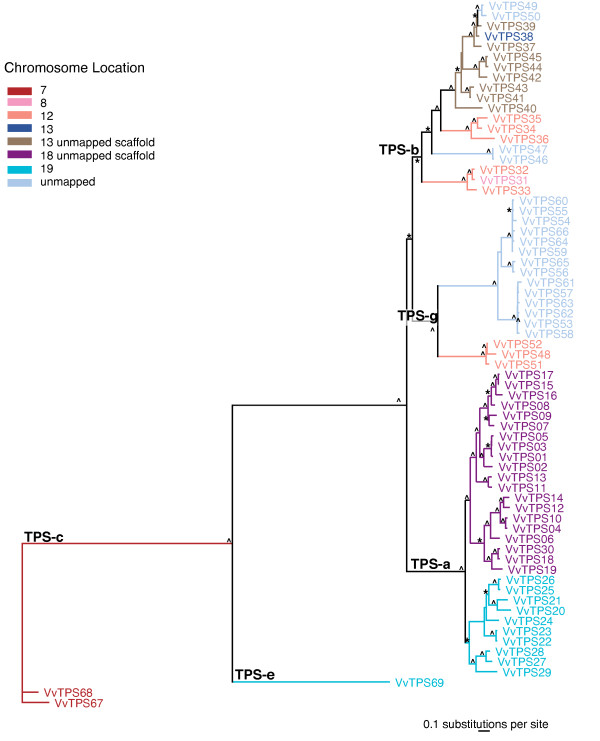
**Phylogeny and chromosome location of 69 putative intact VvTPS identified as gene models in the 12-fold coverage genome sequence assembly of *Vitis vinifera *(Pinot noir)**. Maximum likelihood analysis of the *V. vinifera VvTPS *gene family. Bootstrap values supported by ≥ 50% are designated * and those with values ≥ 80% are indicated with ^. Colors indicate chromosome location known at this time. Light blue = unmapped scaffold, brown = chromosome 13 (unmapped scaffold), dark blue = chromosome 13, orange = chromosome 12, pink = chromosome 8, purple = chromosome 18 (unmapped scaffold), teal = chromosome 19, and red = chromosome 7.

The 69 candidate *VvTPS *sequences identified as intact or potentially intact represent five of the seven plant *TPS *gene subfamilies *TPS-a *through *TPS-g *previously described [16,33,37] (Figure 1; Additional File 1). The *TPS-f *subfamily of *Clarkia brewerii *linalool synthase-like genes and the gymnosperm-specific *TPS-d *subfamily [33], were the only subfamilies missing full-length *VvTPS *members. Although the previous 8-fold grapevine genome assembly [1] contained one *VvTPS-f *subfamily member, in the manually curated assembly and annotation of the 12-fold genome sequence, this gene was now fragmented into two partial *TPS *(Additional File 1).

The *TPS-a *subfamily is substantially extended in grapevine with 30 *VvTPS *existing on just two chromosomes, chromosomes 18 and 19, compared to 22 *AtTPS *of the *TPS-a *subfamily in *A. thaliana *[16]. This subfamily typically contains sesquiterpene synthases and possibly diterpene synthases of secondary metabolism. A total of 19 *VvTPS *were found in the *TPS-b *subfamily of angiosperm monoterpene synthases and these were located on at least three chromosomes, chromosomes 8, 12 and 13. The *TPS-g *subfamily, which contains synthases for acyclic monoterpenes of floral scent [37] is greatly extended in grapevine with 17 *VvTPS *annotated compared to *Arabidopsis *with just one *AtTPS *gene in this subfamily. The chromosomal location of most of the *VvTPS *of the *TPS-g *subfamily is unknown. We found two *VvTPS *of the *TPS-c *subfamily and one *VvTPS *of the *TPS-e *subfamily. These two subfamilies contain *TPS *genes of primary plant hormone metabolism that are not typically represented with multiple gene copies in plant genomes [16,33,38].

### Chromosomal location of *VvTPS*

**Figure 2:**
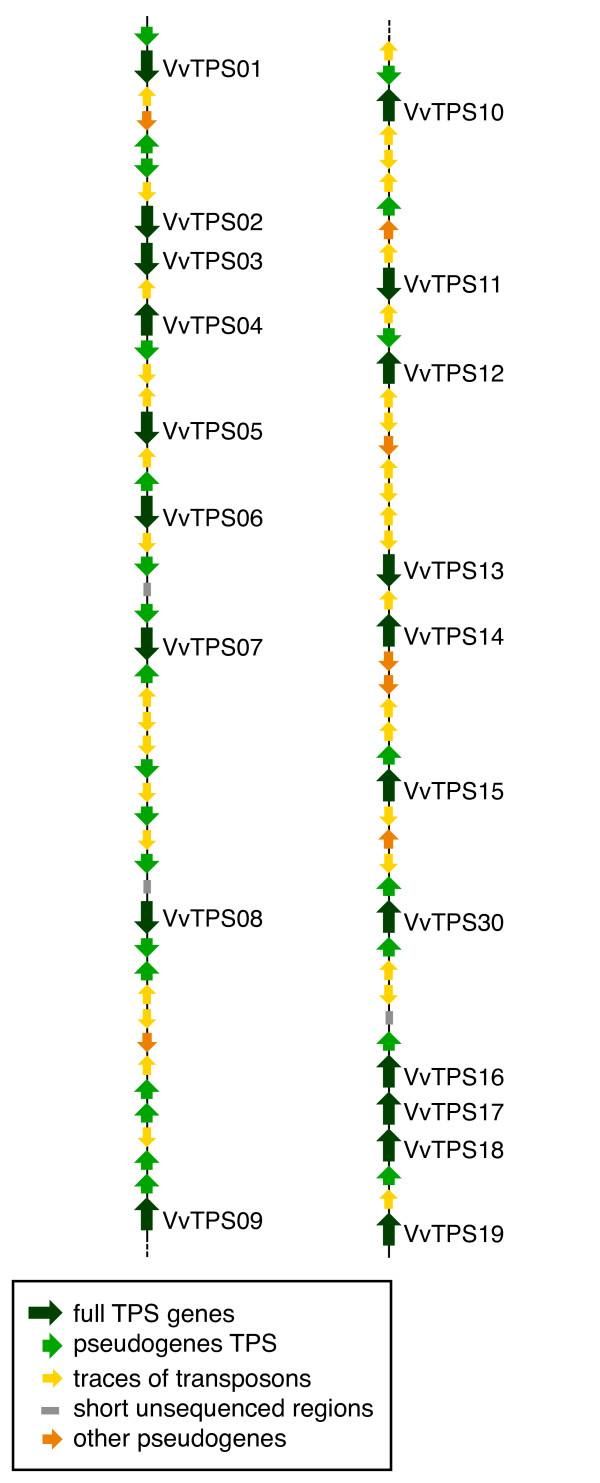
**Genomic organization of a 690 kb multi-gene *VvTPS *cluster**. A 690 kb long genomic region of chromosome 18 contains a large cluster of 20 complete *VvTPS-a *genes (dark green arrows), 25 pseudo-*TPS-a *genes characterized by several deletions, frameshifts and/or stop codons (light green arrows), few traces of other genes (orange arrows) and numerous vestiges of the Copia-like transposable element (yellow arrows).

The topological organization of the *VvTPS *family in the grapevine genome is characterized by massively tandemly repeated genes. Of the complete set of 152 *VvTPS *loci identified in this study, 129 (85%) are organized in 13 distinct clusters covering from 2 to 45 *VvTPS *genes or pseudogenes (Additional File 1). The largest *VvTPS *cluster, localized on chromosome 18 and spanning 690 kb, contains 20 complete *VvTPS *genes (all are members of the *TPS-a *subfamily), 25 *VvTPS *pseudogenes and numerous traces of Copia-like retrotransposons (Figure 2). Although many *VvTPS *cluster together, of the 152 *VvTPS *loci, only 2 *VvTPS *genes also localize in the vicinity of other putative terpenoid pathway genes with the same gene orientation: one (*VvTPS42*) co-localizes with a prenyltranferase gene and the other (the pseudogene GSVIVT01014893001) with a cytochrome P450 gene. Dynamically expanding or contracting clusters of closely related genes can evolve as the result of unequal cross-over, which enriches genetic variability but limits the divergence through an opposing mechanism of gene conversion as has been shown for plant resistance genes [39,40]. These processes can be intensified by the presence of pseudogenes which contribute to the frequency of crossing-over and increase in gene diversity [41]. As previously shown for the *Drosophila melanogaster *genome, a high density of repeat elements can also impact the recombination dynamic within gene clusters [42]. The genome architecture of the *VvTPS *gene family (*i.e.*, the number, size and nature of *VvTPS *clusters in the grapevine genome) suggests a large potential for diversification and variation of terpenoid metabolism in this species, and may thus account for variability of terpenoid profiles among grapevine varieties and cultivars. The identification of *VvTPS *gene clusters allows for future work in which resequencing of these regions in different varieties and testing for associations of gene cluster and terpenoid aroma traits can be undertaken.

### Intron-exon structure of *VvTPS *genes

**Figure 3:**
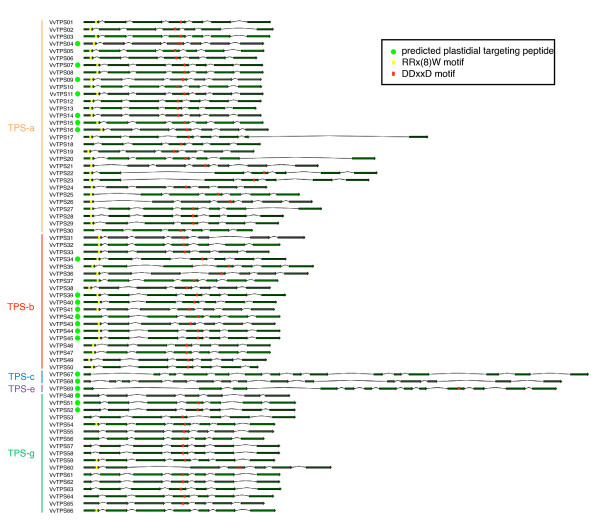
**Gene structure and classification of putative intact VvTPS**. Exon-intron structures were predicted and manually curated for the putative intact *VvTPS *gene models. Green arrows and black lines represent at scale protein coding exons and introns, respectively. The conserved motifs RR(x)_8_W and DDxxD are represented by yellow and red boxes, respectively. Green circles indicate the prediction of an N-terminal plastidial targeting peptide. Classification into subfamilies is based on phylogenetic analyses supported, where applicable, by corresponding functional data. Subfamilies TPS-a, TPS-b and TPS-g are characterized by a highly conserved structure of seven exons. The three *VvTPS *genes of subfamilies TPS-c and TPS-e have distinct structures with 13 to 15 exons.

In agreement with highly conserved intron-exon structure of plant *TPS *genes [16,43,44], all but one of the 66 *VvTPS *genes of the subfamilies *TPS-a*, *TPS-b *and *TPS-g *contain seven coding exons (Figure 3). The only exception is *VvTPS17 *(*TPS-a*) in which the 3'-most exon was disrupted by a large and probably recent intron insertion. The three genes of the subfamilies *TPS-c *and *TPS-e *are characterized by longer sequences (15 and 13 exons respectively) as a consequence of the presence of an additional exon encoding an ancestral 200 amino acid N-terminal domain of unknown function [16,33,38].

### Conserved motifs of the *V. vinifera *TPS protein family

**Figure 4:**
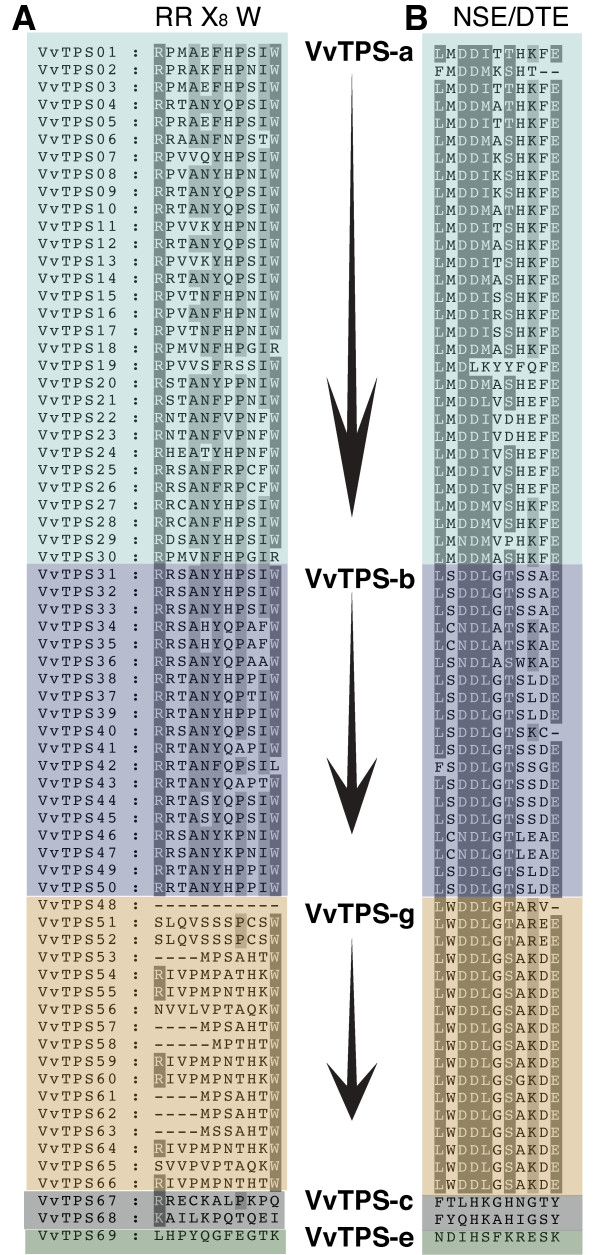
**The conserved RR(X_8_)W and NSE/DTE motifs in VvTPS**. A: Alignment of the RR(x)_8_W motif within the 69 intact VvTPS annotated in this analysis. B: Alignment of the NSE/DTE motif within the 69 intact VvTPS annotated in this analysis. Dark grey shading indicates amino acids conserved with ≥ 80% and those shaded with light grey indicate conservation ≥ 60%.

The grapevine VvTPS protein family is characterized by two large domains defined in the PFAM resource [45]: PF01397 corresponds to the N-terminal region and PF03936 corresponds to the C-terminal metal cofactor binding domain [46]. Just upstream of the PF01397 N-terminal domain, in the region encoded by the first exon, all VvTPS that putatively function as monoterpene synthases, also contain the RR(x)_8_W motif. This motif may play a role in the initiation of the isomerization-cyclization reaction [47] or act to stabilize the protein through electrostatic interactions [48]; however, *TPS *in subfamily *TPS-g*, as well as two VvTPS predicted in *TPS-a*, and those in *TPS-c *and *TPS-e *do not consistently contain this motif or they have a highly modified version of it (Figure 3 and 4A). Several of the *TPS-g *members are also truncated with the starting M at position five of this motif. This may effectively open up the three dimensional structure of these TPS or it may affect subcellular compartmentation of these proteins. Mono- and diterpene synthases typically contain an N-terminal plastidial targeting peptide upstream of the conserved or modified RR(x)_8_W [33], and such targeting peptides have been predicted *in silico *for 21 VvTPS (Figure 3).

The C-terminal domain contains two highly conserved aspartate-rich motifs. The first of these, the DDxxD motif (Figure 3), is involved in the coordination of divalent ion(s), water molecules and the stabilization of the active site [46,49,50]. Only four predicted VvTPS (VvTPS48, VvTPS66 and the two *TPS-c *proteins VvTPS67 and VvTPS68) lack the exact DDxxD motif characteristic of class I TPS which catalyze reactions initiated by cleavage of the diphosphate group of the prenyl diphosphate substrate. The TPS-c proteins are not expected to have this domain as they do not cleave the prenyl diphosphate unit; however, they do contain the DXDD sequence critical to the protonation initiated reaction mechanism of class II TPS [51].

A second important motif in the C-terminal domain is the NSE/DTE motif [52,53]. The reported consensus sequence of this motif is (L,V)(V,L,A)(N,D)D(L,I,V)x(S,T)xxxE and a modified version, (L,F)(M,I,S,C,W)(N,D)D(L,M,I)x(S,T,D)xxxE, is found in almost all VvTPS of the subfamilies *TPS-a*, -*b*, and -*g *(Figure 4B). Three predicted VvTPS are lacking the terminal E. Members of the TPS-g subfamily have an altered and highly conserved sequence, LWDDLx(S,T)xxxE.

### Functional characterization of *VvTPS *full length cDNAs

Since specific functions of *TPS *genes cannot be accurately predicted from sequence analysis alone, it was important to clone and functionally characterize *VvTPS *full length (FL)cDNAs. We used the previously published 8-fold [1] and the new 12-fold genome sequence (GenBank, NCBI project ID 18785) assembly of the Pinot noir inbred line for primer design to clone *VvTPS *FLcDNAs from three grapevine varieties, Pinot noir (PN), Cabernet Sauvignon (CS), and Gewürztraminer (Gw). FLcDNAs were expressed in *E. coli *using one of several cloning vectors (see Materials and Methods), and recombinant VvTPS proteins were functionally characterized via purified protein or *in vivo *recombinant *E. coli *assays with each of the following potential substrates, geranyl diphosphate (C10, GPP), (*E*,*E*)-farnesyl diphosphate (C15, FPP), and (*E*,*E*,*E*)-geranylgeranyl diphosphate (C20, GGPP). Although a couple of recent studies identified two tomato TPS which utilize neryl diphosphate or (*Z*,*Z*)-farnesyl diphosphate as substrates [54,55], these TPS are members of the TPS-e subfamily and none of the VvTPS that we characterized belonged to this subfamily. In addition, a screen of the grapevine genome sequence did not reveal the presence of *Z*-isoprenyl diphosphate synthases (unpublished results, M. Chavez, S. Aubourg, and J. Bohlmann), therefore, we did not include these alternative substrates when assaying for VvTPS activity. Products were analyzed by GCMS and the majority of VvTPS analyzed produced multiple products (Table 1, Table 2, Table 3, and Table 4), which is a common feature of plant TPS [56,57]. Since many of the *VvTPS *FLcDNAs arose from cloning efforts in all three *V. vinifera *varieties PN, CS, and Gw, a number of *VvTPS *were uncovered as cultivar-specific variants, each differing by only a few amino acids. To ensure that these were not unique genes capable of producing a distinct product profile, each variant was functionally characterized. However, only one representative cDNA clone will be described for its function following below, while the additional clones from the other cultivars are listed in Table 1, Table 2, Table 3, and Table 4.

**Table 1:** Experimental Functional Annotation of *VvTPS *Genes of the VvTPS-a Subfamily

Functional Gene ID	Function	Access. #	FLcDNA ID	Nearest VvTPS gene model	Representative Products	%
VvGwECar1	(*E*)-β-Caryophyllene Syn	HM807373	Gw38F6*	VvTPS01	(*E*)-caryophyllene	71
				
VvGwECar2		HM807374	Gw53B1*	VvTPS27	α-humulene	23
				
VvGwECar3		HM807375	Gw12001M3*	VvTPS02	germacrene D	6
				
VvPNECar1		HM807402	CAN82172^	VvTPS02		
				
VvPNECar2		HM807403	CAO16256^	VvTPS13		

VvGwGerA	Germacrene A Syn	HQ326230	Gw38F3*	VvTPS01	germacrene A	52
					α-selinene	24
					selin-11-en-4-α-ol	12

VvGwaBer	(*E*)-α-Bergamotene Syn	HM807376	Gw56B1*	VvTPS10	(*E*)-α-bergamotene	56
					Unknown	17
					Nerolidol	14
					(*E*)-β-farnesene	8
					(*Z*)-α-farnesene	5

VvGwGerD	Germacrene D Syn	HM807377	Gw64B1*	VvTPS07	germacrene D	94
				
VvPNGerD		HM807378	PN39M3*	VvTPS15	germacrene B	6

VvCSaFar	(*E,E*)-α-Farnesene syn	HM807379	CS102B7*	VvTPS20	(*E,E*)-α-farnesene	100

VvGwgCad	γ-Cadinene Syn	HM807380	Gw330M5*	VvTPS08	γ-cadinene	83
					Unknown	17

VvPNbCur	β-Curcumene syn	HM807381	PN62M1*	**VvTPS30**	β-curcumene	22
					(*E*)- γ-bisabolene	18
					Iso-italicene	16
					(-)-α-bisabolol	14
					Β-bisabolene	7
					epi-β-santalene	7
					unknown	5
					(*E*)-β-farnesene	3
					γ-curcumene	3
					unknown	2
					(*Z*)-α-bergomotene	1
					unknown	1

VvPNSesq	Sesquithujene Syn	HM807404	CAO16252^	VvTPS12	(*E*)-α-bergomotene	1
					sesquithujene	80
					(*E*)-α-bergamotene	4
					sesquisabinene	8
					β-bisabolene	4
					γ-bisabolene	2
					unknown pm 222	1.5
					β-bisabolol	0.5

VvPNaZin	α-Zingiberene syn	HM807405	CAO16257^	VvTPS14	α-zingiberene	79.5
					β-sesquiphellandrene	17.5
					β-bisabolene	3

VvPNSeInt	Selina-411-diene/Interme deol syn	HM807406	CAO39293^	VvTPS24	selina-4,11-diene	34
					intermedeol	30
					7-epi-α-selinene	15
					δ-selinene	14
					α-guaiene	3.5
					selina-511-diene	2
					unknown pm 204	1

VvPNCuCad	Cubebol/δ-Cadinene syn	HM807407	CAN76781^	VvTPS26	cubebol	20.5
					δ-cadinene	20
					unknown pm 222	16.5
					α-cubebene	14
					α-copaene	13.5
					α-gurjunene	7.5
					γ-cadinene	3
					β-cubebene	2.5
					unknown pm 204	2.5

VvPNaHum	α-Humulene syn	HM807408	CAN64791^	VvTPS11	α-humulene	56
					hyemalol	37.5
					(*E*)-β-caryophyllene	6.5

VvPNEb2epi Car	(*E*)-β-Caryophyllene/2-epi-(*E*)-β-Caryophyllene	HM807409	CAO39418^	VvTPS21	(*E*)-β-caryophyllene	72.5
					2-epi-(*E*)-β-caryophyllene	25

Terpenoids produced by individual VvTPS clones when incubated with FPP are listed. FLcDNA clones with redundant functions are listed as well. Clones marked with an * were characterized by in vitro assays with isolated recombinant VvTPS; clones marked with ^ were characterized *in vivo *in metabolically engineered *E. coli*. The tissue specific cDNA used to clone a particular gene is indicated in FLcDNA ID with either B (berry), F (flower), or M (mixed template consisting of stems, berries, flowers, and leaves) when appropriate. FLcDNA ID in bold indicates clone came from cDNA of methyl jasmonate treated tissue. Of the clones labelled as (*E*)-β-caryophyllene syn all produced an abundance of (*E*)-β-caryophyllene, with minor components of α-humulene and germacrene d except CAN82172 which did not produce germacrene D. Bolded *VvTPS *gene models indicate proof of transcript not previously known from the available ESTs.

**Table 2:** Experimental Functional Annotation of *VvTPS *Genes of the VvTPS-b Subfamily

Functional Gene ID	Function	Access. #	FLcDNA ID	Nearest VvTPS gene model	Representative Products	%
VvGwaPhe	(+)-α-Phellandrene Syn	HM807382	**Gw74ME**	VvTPS45	(+)-α-phellandrene	40
					myrcene	15
					terpinolene	15
					α-terpinene	7
					γ-terpinene	6
					(+)-limonene	5
					(+)-α-pinene	3
					(+)-β-phellandrene	3
					(+)-α-terpineol	3
					(*E*)-β-ocimene	2

VvPNaPin1	(+)-α-Pinene Syn	HM807383	**PN20M1**	VvTPS44	(+)-α-pinene	47
				
VvPNaPin2		HM807384	**PN05S14**	VvTPS44	(+)-limonene	25
				
					(+)-camphene	9
					myrcene	6
					(+)-α-terpineol	5
					(+)-sabinene	3
					(3*S*)-linalool	2
					(+)-β-pinene	1
					(+)-α-phellandrene	1
					(+)-α-thujene	1
					α-terpinolene	1

VvGwbOci	(*E*)-β-Ocimene syn	HM807385	Gw22YB2	**VvTPS34**	(*E*)-β-ocimene	98
				
VvCSbOci		HM807386	CS402F	VvTPS35	(*Z*)-β-ocimene	2

VvCSbOciM	(*E*)-β-Ocimene/Myrcene syn	HM807387	CS19M	VvTPS38	(*E*)-β-ocimene	53
					myrcene	42
					β-pinene	3
					limonene	2

VvGwbOciF	(*E*)-β-Ocimene/(*EE*)-α-Farnesene Syn	HM807388	Gw46YB3	**VvTPS47**	(*E*)-β-ocimene	100*
				
VvCSbOciF		HM807389	CS93F	**VvTPS47**	(*E,E*)-α-farnesene	100**

VvPNRLin	(*3R*)-Linalool syn	HM807390	**PNTPS09M1**	**VvTPS31**	(*3R*)-linalool	100

Terpenoids produced by individual VvTPS clones when incubated with GPP are listed. Where noted are bifunctional TPS capable of producing products when incubated with GPP (*) or FPP (**). The tissue specific cDNA used to clone a particular gene is indicated in FLcDNA ID with either B (berry), YB (young berry), F (flower), S (stem) or M (mixed template consisting of stems, berries, flowers and leaves). FLcDNA ID in bold indicates clone came from cDNA of methyl jasmonate treated tissue. Clones with redundant functions are listed as well. Bolded *VvTPS *gene models indicate proof of transcript not previously known from the available ESTs.

**Table 3:** Experimental Functional Annotation of *VvTPS *Genes of the VvTPS-g Subfamily

Functional Gene ID	Function	Access. #	FLcDNA ID	Nearest VvTPS gene model	Representative Products	%
VvPNLinNer1	(3*S*)- Linalool/(*E*)- Nerolidol syn	HM807391	**PN25M6**	VvTPS54	(3*S*)-Linalool	100*
				
VvPNLinNer2		HM807392	**PN55M1**	VvTPS56		
				
VvCSLinNer		HM807393	CS2251F	VvTPS56	(*E*)-Nerolidol	100**

VvPNLNGl1	Linalool/(*E*)- Nerolidol/(*E,E*)-Geranyl linalool syn	HM807394	**PNTPS271M2**	**VvTPS57**	(3*S*)-Linalool	100*
				
VvPNLNGl2		HM807395	**PNTPS271M5**	**VvTPS63**	(*E*)-Nerolidol	100**
				
VvPNLNGl3		HM807396	**PNTPS271M4**	VvTPS58	(*E,E*)-Geranyl linalool	100***
				
VvPNLNGl4		HM807397	**PN3M2**	**VvTPS61**		

VvGwGer	Geraniol syn	HM807398	Gw63YB3	VvTPS52	Geraniol	100
				
VvCSGer		HQ326231	**CS5M2**	VvTPS52		
				
VvPNGer		HM807399	**PN5L1**	VvTPS52		

Terpenoids produced by individual VvTPS clones when incubated with GPP (*) or FPP (**) are listed. Where noted are trifunctional TPS capable of producing products also with GGPP (***). The tissue specific cDNA used to clone a particular gene is indicated in FLcDNA ID with either YB (young berry), F (flower), L (leaves) or M (mixed template consisting of stems, berries, flowers and leaves). FLcDNA ID in bold indicates clone came from cDNA of methyl jasmonate treated tissue. Clones with redundant functions are listed. Bolded *VvTPS *gene models indicate proof of transcript not previously known from the available ESTs.

**Table 4:** Experimental Functional Annotation of *VvTPS *Genes of the VvTPS-f Subfamily

Functional Gene ID	Function	Access. #	FLcDNA ID	Nearest VvTPS gene model	Representative Product Profile	%
VvCSENerGl	(*E*)-Nerolidol/(*E,E*)-Geranyl linalool syn	HM807400	CS34137F	NA	(*E*)-Nerolidol	100*
				
VvPNENerGl		HM807401	PNTPS51F2	NA	(*E,E*)-Geranyl linalool	100**

Terpenoids produced by individual VvTPS clones when incubated with FPP (*) or GGPP (**) are listed. The tissue specific cDNA used to clone a particular gene is indicated in FLcDNA ID with F (flower). Clones with redundant functions are listed also.

A subset of *VvTPS *FLcDNAs of the *TPS-a *subfamily (Table 1) were chemically synthesized and characterized using an *E. coli *strain engineered to produce the FPP substrate from mevalonate. Based on previous work [58], an operon encoding the mevalonate lower pathway of *Streptoccoccus pneumoniae *was subcloned into a bacterial expression vector together with the *Saccharomyces cerevisiae *FPP synthase. *VvTPS *FLcDNAs were additionally expressed into this engineered strain and product formation was measured by GCMS in the culture extract as has been done for the characterization of other TPS and cytochrome P450 s [59].

### Nomenclature for functionally characterized *VvTPS *FLcDNAs

We assigned gene identifiers that include references to both function and the cultivar (PN, CS, or Gw) from which the gene was isolated (see Functional Gene ID in Table 1, Table 2, Table 3, and Table 4). These functional gene identifiers will be used throughout the following sections to describe individual genes. Table 1, Table 2, Table 3, and Table 4 provide additional detailed information for each FLcDNA regarding clone ID, tissue origin (see table legend), product profiles with relative quantitative information, as well as identification of the closest annotated *VvTPS *gene model reported in this paper. In some instances multiple cDNAs share the same functional gene identifier, but are represented as distinct genes because they occupy unique locations within the *VvTPS *phylogeny. These functional gene IDs are designated with numbers in both the tables and within the phylogenetic trees.

### Functions of *VvTPS *FLcDNAs of the TPS-a Subfamily

The majority of *VvTPS *genes belong to the TPS-a subfamily for which we functionally characterized 13 unique FLcDNAs (Table 1). All of the *VvTPS-a *members were characterized as sesquiterpene synthases, and all but one formed multiple products with FPP as substrate. In several cases, the product profiles included both terpenoid hydrocarbons and alcohols. As a group, the VvTPS of the *TPS-a *subfamily produce a diverse array of sesquiterpene products.

All five individual VvGwECar and VvPNECar FLcDNA clones produced predominantly (*E*)-β-caryophyllene. Four of these clones also produced α-humulene and a small amount of germacrene D, while one clone (CAN82172) produced only (*E*)-β-caryophyllene (94%) and α-humulene (6%). The two VvPNECar enzymes characterized in metabolically engineered *E. coli *(clones CAO16256 and CAN82172) showed similar product profiles to VvGwECar clones characterized by *in vitro *enzyme assays. One of the VvPNECar enzyme (CAO16256) also produced a low amount ( < 1%) of an unknown sesquiterpene alcohol in addition to (*E*)-β-caryophyllene, α-humulene and germacrene D. The TPS VvGwGerA (Gw38F3) produced primarily germacrene A (52%) and α-selinene (24%) and a small amount of selin-11-en-4-α-ol (12%). The product profile of VvGwaBer (Gw56B1) consisted of (*E*)-α-bergamotene (56%), zingiberenol (17%) and nerolidol (14%) as well as two minor compounds. Germacrene D was the primary product of VvGwGerD (Gw64B1). VvCSAFar (CS102B7) was the only single-product member of the *TPS-a *subfamily identified here, producing 100% (*E,E*)-α-farnesene. Two products, γ-cadinene (83%) and an unidentified sesquiterpene (17%), were the only detected products of VvGwgCad (Gw330M5). VvPNbCur (PN62M1) produced β-curcumene (22%) (E)-γ-bisabolene (18%), iso-italicene) (16%), (-)-α-bisabolol (14%), and at least 9 additional products. The VvPNSesq (CAO16252) generated a sesquiterpene olefin as major product that was not identified unambiguously by GCMS. The product was, therefore, produced in larger quantity, purified by preparative GC and identified by NMR spectroscopy as sesquithujene ((1 S,5S)-2-methyl-5-((R)-6-methylhept-5-en-2-yl)bicyclo[3.1.0]hex-2-ene). Additional reaction products of this TPS included α-bergamotene, sesquisabinene, β- and γ-bisabolene, β-bisabolol and a trace amount of an unidentified sequiterpene alcohol. VvPNaZin (CAO16257) is a zingiberene synthase that also produced β-sesquiphellandrene and β-bisabolene. The two major products generated by VvPNSeInt (CAO39293) were found to be selina 4,11-diene and intermedeol. At least, five other sesquiterpenes including α-guaiene, selina 5,11-diene, γ-selinene, and 7-epi-α-selinene, were identified as reaction products. VvPNCuCAD (CAN76781) encodes a multi-product sesquiterpene synthase capable of producing cubebol, δ-cadinene, α-copaene, α-cubebene and an unknown sesquiterpene alcohol as dominant reaction products; additional minor products included α-gurjunene, γ-cadinene, β-cubebene and an unknown sesquiterpene. The VvPNaHum (CAN64791) produced α-humulene (56%), (*E*)- β-caryophyllene (6.5%) and a sesquiterpene alcohol (37.5%) that we initially failed to identify by GCMS; the latter compound was then produced in milligram quantities, purified by liquid chromatography and identified by ^1^H- and ^13^C-NMR spectroscopy as hyemalol (3,7,10,10-tetramethylcycloundeca-3,7-dien-1-ol) a humulane-type sesquiterpenoid recently discovered in *Zanthoxylum hyemale *[60]. VvPNEb/2epiCar (CAO39418), was identified as a third type of caryophyllene synthase, which in contrast to VvPNECar and VvGwECar also catalyzed the formation of 2-epi-(*E*)-β-caryophyllene as a substantial (25%) product.

Collectively, these characterized *VvTPS-a *enzymes produce some of the major sesquiterpenes identified from grapevine. Furthermore, the prevalence of VvTPS producing (*E*)-β-caryophyllene, α-humulene and germacrene D may in part explain the reported prominence of these compounds in grapevine berries and vegetative tissues [12-15].

### Functions of *VvTPS *FLcDNAs of the *TPS-b *subfamily

We functionally characterized seven unique *VvTPS *(nine different FLcDNA clones) from the *TPS-b *subfamily (Table 2). All of the characterized *VvTPS *genes of this group produce monoterpenes, and most are multi-product enzymes. The products of VvGwaPhe included (+)-α-phellandrene (40%), myrcene (15%), terpinolene (15%), and seven other minor products. VvPNaPIN produced (+)-α-pinene (47%), (+)-limonene (25%), (+)-camphene (9%), and eight other minor products. Five of the nine VvTPS of the *TPS-b *subfamily (VvGwbOci, VvCSbOci, VvCSbOciM, VvGwbOciF, VvCSbOciF) produced (*E*)-β-ocimene as a major product with individual variations of additional products. VvGwbOci and VvCSbOci produced additional minor amounts of (*Z*)-β-ocimene; VvCSbOciM produced additional major amounts of myrcene (42%) along with minor amounts of β-pinene and limonene; VvGwbOciF and VvCSbOciF also converted FPP into (*E,E*)-α-farnesene. Lastly, VvPNRLin produced a single oxygenated product, (*3R*)-linalool. Together the *VvTPS-b *subfamily members account for many of the acyclic and cyclic monoterpene hydrocarbons and a few of the monoterpene alcohols found in *Vitis vinifera*.

### Functions of *VvTPS *FLcDNAs of the TPS-g subfamily

The TPS-g subfamily is greatly expanded in *V. vinifera *(Figure 2, Addiitonal File 1). Functional characterization of ten different FLcDNA clones of this subfamily identified three unique gene functions (Table 3). All VvTPS of this group produce exclusively acyclic terpene alcohols, but the three types differ by their range of substrates. The first type of *VvTPS *gene function within the TPS-g subfamily is represented by three genes, VvPNLinNer1, VvPNLinNer2, and VvCSLinNer. These enzymes accept two substrates, C10 GPP and C15 FPP, and produce (3*S*)-linalool and (*E*)-nerolidol, respectively (Table 3). The second group is represented by four VvPNLNGl enzymes which also accept the additional C20 substrate GGPP to produce (*E*,*E*)-geranyl linalool. The third unique function in this subfamily is represented by three genes, VvGwGer, VvCSGer and VvPNGer, which had activity only with GPP to produce geraniol. While seven of the VvTPS of the *TPS-g *subfamily accept more than one substrate *in vitro *and contribute potentially to the formation of terpene alcohols of different chain lengths, it is not known whether these enzymes indeed encounter more than one type of substrate *in vivo*.

### Functions of *VvTPS *FLcDNAs of the TPS-f subfamily

Although the analysis of the 12-fold genome sequence coverage did not identify any intact *VvTPS *genes of the TPS-f subfamily, a unique VvTPS function of the *TPS-f *subfamily was characterized with the two FLcDNAs VvCSENerGl and VvPNENerGl (Table 4). These enzymes accepted either FPP or GGPP to produce (*E*)-nerolidol or (*E*,*E*)-geranyl linalool, respectively. Unlike the VvTPS of the TPS-g these enzymes had no activity with GPP. VvCSENerGl and VvPNENerGl are only 62% identical and 76% similar on an amino acid level.

### Phylogeny of functionally characterized VvTPS and VvTPS gene models

The phylogenetic analyses presented here include *V. vinifera *TPS from the 12-fold sequence assembly of the nearly homozygous Pinot noir genotype [1] and the functionally characterized VvTPS described here. The analyses also included full-length TPS sequences that contained the known TPS motifs predicted from the genome assembly of the heterozygous Pinot noir genotype [36] for a more complete annotation of the *VvTPS *family. In this way, we have integrated the predictions of *VvTPS *gene models from the two grapevine genome sequences [1,36] in a compatible fashion and we are proposing a unified *VvTPS *classification and nomenclature.

**Figure 5:**
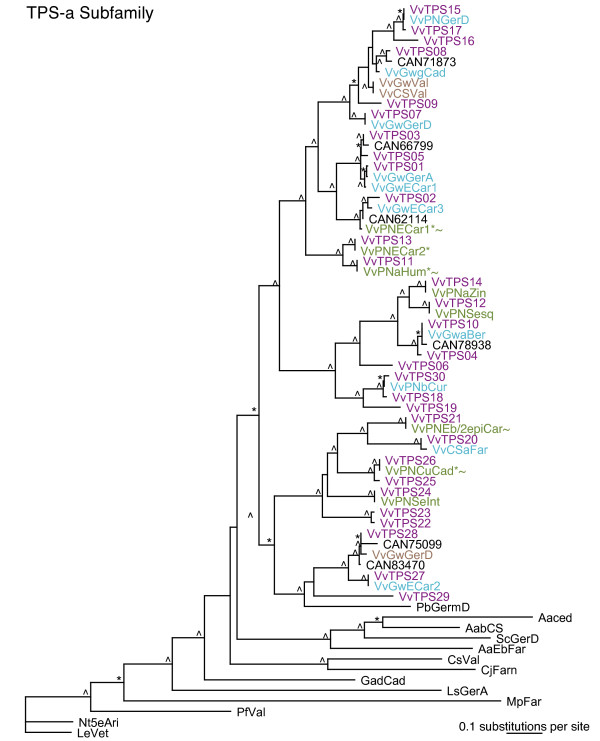
**Phylogeny of the VvTPS-a subfamily**. Maximum likelihood analysis of the *V. vinifera *TPS-a subfamily. Bootstrap values supported by ≥ 50% are designated * and those with values ≥ 80%are indicated with ^. TPS characterized in this paper are in teal and include Vv(PN & Gw)ECar# = (*E*)-caryophyllene syn, VvPNGerA = germacrene A syn, VvGwaBer = (*E*)-α-bergamotene syn, Vv(PN & Gw)GerD = germacrene D syn, VvCSaFar = (*E*,*E*)-α-farnesene syn, VvGwgCad = γ-cadinene syn, VvPNbCur = β-curcumene syn, VvPNSesq = sesquithujene syn, VvPNaZin = α-zingiberene syn, VvPNSeInt = Selina-4,11-diene/Intermedeol syn, VvPNCuCad = cubebol/δ-cadinene syn, VvPNaHum = α-humulene syn, and VvPNEb2epiCar = (*E*)-β-caryophyllene/2 epi-(*E*)-β-caryophyllene syn. VvTPS predicted from the 12-fold genome sequence assembly are in purple. Previously cloned VvTPS are in brown. TPS predicted by sequencing of the heterozygous Pinot noir are labeled with GenBank accession numbers (CAN....) or marked with ~ if they were also cloned and characterized. Abbreviations are as follows: AabCS = *Artemisia annua*, β-caryophyllene syn (AAL79181), VvGwGerD = *Vitis vinifera *(-)-germacrene D syn (AAS66357), VvCsVal = *V. vinifera *(+)-valencene syn (ACO36239), Aaced = *A. annua *8-epi-cedrol syn (AAF80333), ScGerD = *Solidago canadensis *(-)-germacrene D syn (AAR31145), AaEbFar = *A. annua *(*E*)-β-farnesene syn (AAX39387), CjFarn = *Citrus junos *(*E*)-β-farnesene syn (AAK54279), LsGerA = *Lactuca sativa *germacrene A syn (AAM11627), LeVet = *Lycopersicon esculentum *vetispiradiene syn (AAG09949), GadCad = *Gossypium arboreum *(+)-δ-cadinene syn (O49853), PfVal = *Perilla frutescens var. frutescens *valencene syn (AAX16077), CsVal = *C. sinensis *valencene syn (AAQ04608), Nt5eAri = *Nicotiana attenuata *5-epi-aristolochene syn (AAP05761), MpFar = *Mentha x piperita *(*E*)-β-farnesene syn (AAB95209).

Within the TPS-a subfamily of sesquiterpene synthases the functionally characterized VvTPS are close to most of the *VvTPS *predicted in the 12-fold genome sequence assembly (Figure 5). This topology suggests that the diversity of functions for grapevine sesquiterpene synthases is well represented with the functionally characterized VvTPS described in this work. Relative to TPS-a enzymes of other plant species, the *VvTPS *exhibit a large paralogous cluster with *VvTPS-a *members more closely related to one another than they are to TPS from other species, regardless of function. Paralogous TPS gene clusters were found previously for other species examined in depth such as *A. thaliana *[16] and Norway spruce [56] and indicate post-speciation gene duplication events. The large number of *VvTPS-a *suggests that this subfamily plays an important role in grapevine biology.

**Figure 6:**
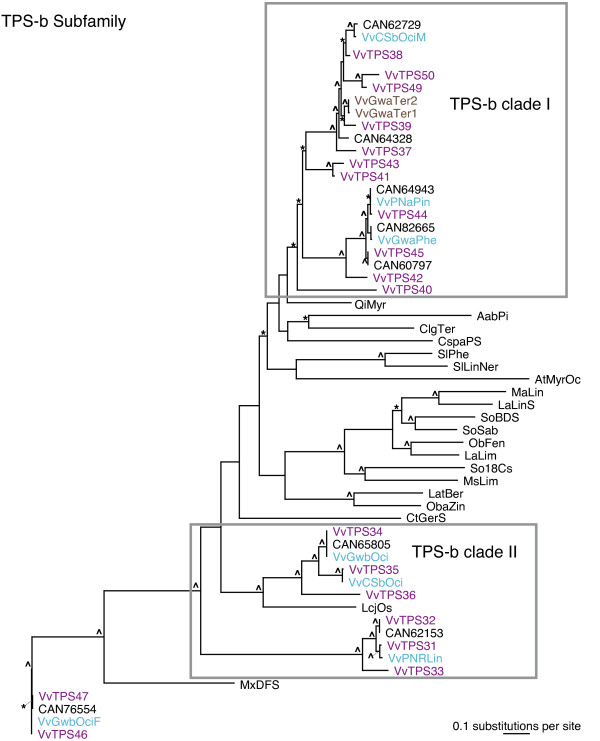
**Phylogeny of the TPS-b subfamily**. Maximum likelihood analysis of the *V. vinifera *TPS-b subfamily. Bootstrap values supported by ≥ 50% are designated * and those with values ≥ 80%are indicated with ^. TPS characterized in this paper are in teal and include VvGwPhe = (+)-α-phellandrene syn, VvPNpPin = (+)-α-pinene syn, VvGwbOci &VvCSbOci = (*E*)-β-ocimene syn, VvCSEbOcM = (*E*)-β-ocimene/myrcene syn, VvGwEbOciF = (*E*)-β-ocimene/(*E*,*E*)-α-farnesene syn, and VvPNRLin = (*3R*)-linalool syn. VvTPS predicted from the 12-fold genome sequence assembly are in purple. Previously cloned VvTPS are in brown. TPS predicted by sequencing of the heterozygous Pinot noir are labeled with GenBank accession numbers (CAN....). Abbreviations are as follows: CspaPS = *Cannabis sativa *(+)-α-pinene syn (ABI21838), LaRLin = *Lavandula angustifolia *(*3R*)-linalool syn (ABD77417), CtGerS = *Cinnamomum tenuipile *geraniol syn (CAD29734), VvaTer1 = *V. vinifera *(-)-α-terpineol syn (AAS79351), VvaTer2 = *V. vinifera *(-)-α-terpineol syn (AAS79352), QiMyr = *Quercus ilex *myrcene syn (Q93X23), ClgTer = *C. limon *γ-terpinene syn (AAM53943), LaLim = *L. angustifolia *(+)-limonene syn (ABB73044), SoSab = *Salvia officinalis *(+)-sabinene syn (O81193), MxDFS = *Malus x domestica *(*E*,*E*)-α-farnesene syn (AAX19772), AabPi = *A. annua *(-)-β-pinene syn (AAK58723), MsLim = *Mentha spicata *(-)-limonene syn (AAC37366), AtMyrOc = *Arabidopsis thaliana *myrcene/(*E*)-β-ocimene syn (NP_179998), MaLin = *M. aquatica *(*3R*)-linalool syn (AAL99381), SoBDS = *S. officinalis *(+)-bornyl diphosphate syn (O81192), So18Cs = *S. officinalis *1,8-cineole syn (O81191), LcjOs = *Lotus corniculatus var. japonicus *(*E*)-β-ocimene syn (AAT86042), ObaZin = *Ocimum basilicum *α-zingiberene syn (AAV63788), LatBer = *L. angustifolia *(*E*)-α-bergamotene syn (ABB73046), ObFen = *O. basilicum *fenchol syn (AAV63790), SlLinNer = *Solanum lycopersicum *(*3R*)-linalool/(*E*)-nerolidol syn (AAX69063), SlPhe = *S. lycopersicum *β-phellandrene/myrcene/sabinene syn (AAX69064)

*VvTPS *of the *TPS-b *subfamily fall into two clades, *VvTPS-b *clade I and *VvTPS-b *clade II, bifurcated by representative TPS from other plants (Figure 6). The majority of the *VvTPS *of clade I make cyclic products while those of clade II produce only acyclic terpenoids. It is possible that clade-specific conserved sequence features determine whether a TPS is able to produce cyclic or acyclic products [53]; thus, the two clades may represent an evolutionary pattern of sub-functionalization from cyclic-product TPS in clade I to those TPS producing acyclic products in clade II. In contrast to other *TPS *subfamilies, the *VvTPS *clades of the *TPS-b *subfamily include members that have functional equivalents in distantly related species. For example, *Lotus japonica *(*E*)-β-ocimene synthase clusters closely with grapevine ocimene synthases. *Malus x domestica *(*E*,*E*)-α-farnesene synthases also clusters closest to VvTPS of the same function. This pattern suggests that these functions arose prior to speciation events.

**Figure 7:**
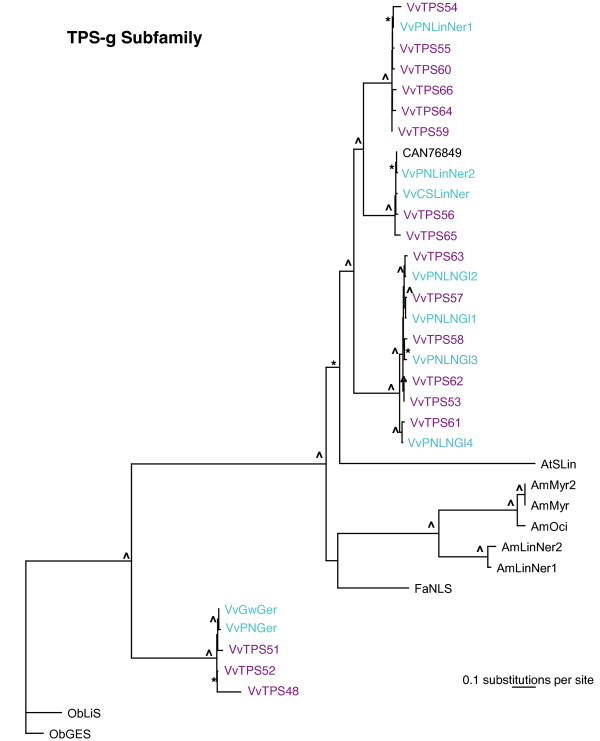
**Phylogeny of the VvTPS-g subfamily**. Maximum likelihood phylogenies of the *V. vinifera *TPS-g subfamily. Bootstrap values supported by ≥ 50% are designated * and those with values ≥ 80%are indicated with ^. TPS characterized in this paper are in teal and include VvPNLinNer1-5 = (3*S*)-linalool/(*E*)-nerolidol syn, VvPNLNGl = (3*S*)-linalool/(*E*)-nerolidol/(*E*,*E*)-geraniol linalool syn, and VvGwGer = geraniol syn. VvTPS predicted from the 12-fold genome sequence assembly are in purple. TPS predicted by sequencing of the heterozygous Pinot noir are labeled with GenBank accession numbers (CAN....). Abbreviations for other tree members are as follows: AmMyr = *Antirrhinum majus *myrcene syn (AAO41727), AmMyr2 = *A. majus *myrcene syn (AAO41726), AmOci = *A. majus *(*E*)-β-ocimene syn (AAO42614.1), FaNLS = *Fragaria x ananassa *(*3S*)-linalool/(*E*)-nerolidol syn (CAD57106), AmLinNer1 = *A. majus *nerolidol/(*3S*)-linalool syn 1 (ABR24417), AmLinNer2 = *A. majus *nerolidol/(*3S*)-linalool syn 2 (ABR24418), AtSLin = *A. thaliana *(*3S*)-linalool syn (NP_176361), ObGES = *O. basilicum *geraniol syn (AAR11765), OCLiS = *O. basilicum *(*3R*)-linalool syn (AAV63789).

The *TPS-g *subfamily of plant *TPS *was defined by previous work on *TPS *of floral scent biosynthesis in snapdragon (*Anthirrrhinum majus*) [37]. Phylogenetic analyses that include the large number of *VvTPS *gene (Figure 1) conclusively resolved a bifurcation of the *TPS-b *and *TPS-g *subfamilies at a juncture that was previously ambiguous and had misclassified some *TPS-g *genes as *TPS-b *members. Specifically, the newly characterized grapevine geraniol synthase VvPNGer which matches gene model VvTPS52 (Figure 1) as well as the geraniol and linalool synthases from basil (*Ocimum basilicum*) (Figure 7), clustered closely with the *TPS-g *subfamily. The phylogenetic proximity between the basil and grapevine geraniol synthases indicates that these TPS functions already existed in a common ancestor. In contrast, the remaining *VvTPS *of the *TPS-g *subfamily, which are all linalool/nerolidol synthases, cluster closest to other *VvTPS*. As mentioned above, the entire predicted *VvTPS-g *subfamily has a conserved NSF/DTE motif (Figure 4). This same motif is present in the cloned *VvTPS *cDNAs as well as other members of the *TPS-g *subfamily from different species. Prominent in this motif of the *VvTPS-g *members is a W in the second position; this residue may affect the magnesium binding and/or substrate orientation. Also noteworthy is the highly modified or absent RRX_8_W motif from this group of TPS (Figure 4) and which may imply that these acyclic products are formed via the geranyl cation rather than the linalyl cation [37,53].

**Figure 8:**
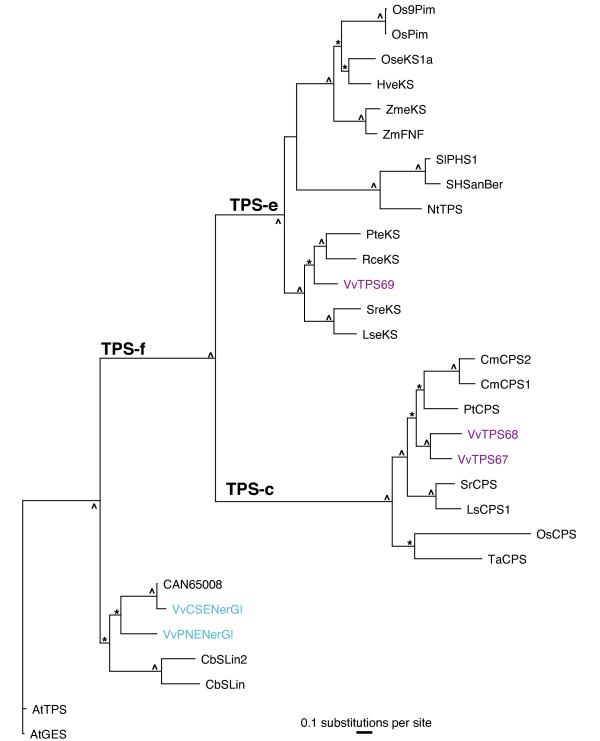
**Phylogeny of TPS-c, e, and f subfamilies**. Phylogenetic relationships as determined by maximum likelihood analysis of the *V. vinifera *TPS-c,e, and -f subfamilies. Bootstrap values supported by *≥ *50% are designated * and those with values ≥ 80% are indicated with ^. TPS characterized in this paper are in teal and include VvCSENerGl and VvPNENerGl = (*E*)-nerolidol/(*E*,*E*)-geraniol linalool syn. VvTPS predicted from the 12-fold genome sequence assembly are in purple. TPS predicted by sequencing of the heterozygous Pinot noir are labeled with GenBank accession numbers (CAN....). TPS-c are all ent-copalyl diphosphate synthases - PtCPS = *Populus trichocarpa *(EEE81383), CmCPS1 = *Cucurbita maxima *(AAD04292), LsCPS1 = *Lactuca sativa *(BAB12440), SrCPS = *Stevia rebaudiana *(AAB87091), CmCPS2 = *C. maxima *(AAD04293), TaCPS = *Triticum aestivum *(BAH56560), OsCPS = *Oryza sativa Japonica group *(BAD42452). The following ent-kaurene synthases (Tps-e) are included in the analysis: PteKS = *Populus trichocarpa *(EEE88653), RceKS = *Ricinus communis *(EEF28689), HveKS = *Hordeum vulgare subsp. Vulgare *(AAT49066), LseKS1 = *Lactuca sativa *(BAB12441), SreKS = *Stevia rebaudiana *(AAD34295), ZmeKS = *Zea mays *(NP_001148059), OseKS1a = *Oryza sativa *Japonica group (AAQ72559). Abbreviations of the other included TPS are as follows: ZmFNF = *Z. mays *(*E*)-β-farnesene, (*E*)-nerolidol, (*E*,*E*)-farnesol syn (AAO18435), OsPim = *O. sativa *Indica group syn-pimara-7,15-diene syn (AAU05906), OS9Pim = *O. sativa *Japonica group 9b-pimara-7,15-diene syn (BAD54751), SHSanBer = *Solanum habrochaites *santalene/bergamotene syn (ACJ38409), SlPHS1 = *S. lycopersicum *β-phellandrene syn (ACO56896), NtTPS = *Nicotiana tabacum *terpene syn (AAS98912), AtGES = *Arabidopsis thaliana *geranyl linalool syn (NP_564772), CbSLin = *Clarkia breweri *(3*S*)-linalool syn (AAC49395), CbSLin2 = *C. breweri *(3*S*)-linalool syn 2 (AAD19840), AtTPS = *A. thaliana *tps (AAO85540).

The *TPS-e *and *TPS-c *subfamilies in *V. vinifera *contain one and two members, respectively (Figure 8). Although these were not functionally characterized in this paper, they are almost certainly involved as diterpene synthases in *ent*-kaurene biosynthesis [61]. Surprisingly, the 12-fold sequence coverage of the grapevine genome did not reveal any members of the TPS-f subfamily; however, our FLcDNA cloning identified two members of this subfamily and both were characterized as nerolidol/geranyl linalool synthases (Figure 8). These are related to the *Clarkia brewerii *linalool synthases [62] and the recently characterized *A. thaliana *geranyl linalool synthase [20], each of which produces acyclic terpene alcohols as a functional signature of this subfamily.

## Conclusions

The present study provides the first comprehensive annotation of the very large *VvTPS *gene family with regard to chromosomal localization, enzyme functions, and phylogeny relative to the overall plant *TPS *gene family. The *VvTPS *gene family is one of the largest gene families of specialized (*i.e.*, secondary) metabolism in grapevine where TPS enzymes contribute to berry and wine flavour, floral scent and potentially a diversity of other biological functions such as defense and resistance. The emerging profile of the *VvTPS *family described here illustrates how this large gene family has expanded across the genome through gene duplication events and functional diversification. Notably, the large number of functionally diverse sesquiterpene synthases identified in our biochemical characterization of the *VvTPS-a *genes suggests that these enzymes and their products may contribute substantially to grapevine biology and wine quality. The recent reports of sesquiterpenes in Shiraz grapes and wine [10,11] or the identification of sesquiterpene volatiles in anthers and pollen of grapevine flowers [32] are early insights to the emerging roles for sesquiterpene metabolism in *V. vinifera*.

Phylogenetic analyses of the *VvTPS *show a result similar to many plant species studied thus far in that most of the *VvTPS *form clusters of paralogous genes within the plant *TPS *family. This finding indicates a dominant process of post-speciation gene duplications, although there are also examples of conserved *TPS *functions of a more ancient order. Furthermore, our analyses substantiate separation of the *TPS-b *and the *TPS-g *subfamilies. The separation based on sequence relatedness is matched by separation of gene functions, since all known members of the *TPS-g *subfamily produce acyclic products.

Of the monoterpene synthases presented here, those that produce (3*S*)-linalool (*VvTPS-g*), geraniol (*VvTPS-g*), and the previously identified α-terpineol synthase (*VvTPS-b*) will be of much interest to viticulturalists and wine makers as these are some of the most prevalent compounds responsible for the floral characteristics of aromatic varieties. Furthermore, compounds such as geraniol and linalool can be further modified in grape musts and wine to produce citronellol, rose oxide, and wine lactone [63,64]. Linalool and α-terpineol have also been found to contribute to the character of non-aromatic red grapevine varieties [65]. It is conceivable that different viticultural regimes may modify the expression of these *TPS *in grape berries and can thereby impact the quality of the resultant wines.

While many of the terpenoid products of the VvTPS enzymes characterized here have been described in the viticulture and oenology literature [8,66], still several have yet to be associated with traits in grapes or wine. Taken together, the present *VvTPS *genomic annotations and the VvTPS functional characterizations provide a reference framework for future studies, including transcript and protein expression profiling, as well as terpenoid molecular marker development through, for example association mapping.

## Methods

### *VvTPS *gene discovery and manual annotation

The predicted proteome of the 12-fold coverage grapevine genome sequence assembly (GenBank, NCBI project ID 18785; Genoscope website: http://www.genoscope.cns.fr/externe/GenomeBrowser/Vitis/) was screened with two HMM profiles of the PFAM motifs [45] PF01397 (N-terminal TPS domain) and PF03936 (TPS, metal binding domain). In addition, the 12-fold genome sequence assembly was screened (TBLASTN) with known TPS sequences from Swiss-Prot in order to be not dependent of the automatic annotation. The 152 loci exhibiting significant similarities with known TPS (all BLAST hits with an e-value lower than 1.e-4 were individually evaluated) were manually annotated to correct erroneous automatic annotation and to discriminate between complete, partial and pseudo-TPS. Genomic regions with similarities spanning on less than 50 amino acids with TPS have not been considered. The manual annotation is based on the results of the EuGène predictor-combiner software [67] that was specifically trained for *Vitis vinifera*, sequence alignments with previously characterized TPS proteins and related PFAM motifs, spliced alignments [68] of cognate EST and cDNA sequences and knowledge of TPS gene structure and protein sequences. Data and other related information were imported and merged in the ARTEMIS tool [69] to evaluate each resource and produce the final annotation. The EuGène predictions, the manual structural annotation of the 152 loci and the corresponding sequences are available in the FLAGdb^++ ^database http://urgv.evry.inra.fr/FLAGdb[70]. Protein sequences deduced from the 69 full *VvTPS *genes were analyzed with ChloroP for prediction of N-terminal plastidial targeting peptides [71]

### Phylogenetic Analyses

Amino acid alignments were made using Dialign (dialign.gobics.de/anchor/submission.php) with a threshold value of 10. Manual adjustments such as aligning conserved motifs and manual trimming were performed using GeneDoc http://www.nrbsc.org/gfx/genedoc. For all analyses, sequence information upstream of the partially conserved RR(X)_8_W motif was trimmed. Maximum likelihood analyses were completed using Phyml [72] available at http://www.atgc-montpellier.fr/phyml/. For each analysis, the LG amino acid substitution model and four substitution rate categories were used, the proportion of invariable sites and the gamma distribution parameter were estimated, and the branch lengths and tree topology were optimized from the data set. The estimated values for the proportion of invariable sites and the gamma shape parameter were then used when performing 100 bootstrap replicas. Phylogenetic trees were visualized using TreeView http://taxonomy.zoology.gla.ac.uk/rod/treeview.html.

### RNA isolation and *VvTPS *cDNA cloning

RNA was isolated from Gewürztraminer, Pinot noir, and Cabernet Sauvignon grapevine shoot cuttings grown in the greenhouse as previously described [32]. RNA was isolated from stem (S), leaf (L), berry (B), root (R) and flower (F) tissues (see FLcDNA ID in Table 1, Table 2, Table 3, and Table 4) as detailed in Reid *et al. *[73]. To up-regulate expression of *TPS *genes, a subset of grapevine cuttings were treated with methyl jasmonate (0.01% v/v in water and 0.1% Tween) two days prior to RNA isolation. The Superscript Vilo cDNA synthesis kit (http://www.invitrogen.com was used according to the manufacturer's instructions. Primers for *VvTPS *cDNA cloning were designed based on TPS sequences obtained through iterative BLAST searches in NCBI GenBank using members of each of the TPS subfamilies. Additional information for the design of PCR primers came from the predicted *VvTPS *gene models identified in the 12-fold genome sequence assembly. To increase the likelihood of a successful PCR amplification of *VvTPS *cDNAs, cDNA templates from various tissues from the same cultivar were often combined. These clones are designated "M" for mixed template while those *TPS *cloned from individual tissues are labeled with the single letter abbreviations described above (Table 1, Table 2, Table 3, and Table 4). PCR reactions were done using touchdown PCR with proofreading polymerases, as per the product manufacturer's instructions. PCR products of the expected sizes were cloned into the pJet1.2 cloning vector http://www.fermentas.com and transformed into *E. coli *α-select cells http://www.bioline.com. Plasmids containing a correctly sized insert were sequenced followed by insert amplification and ligation into the pET28b expression vector (Novagen, http://www.emdchemicals.com) using sticky end PCR [74]. When cloning difficulties were encountered, as was the case with several sequences, pASK-IBA3plus (IBA, http://www.iba-go.com) vector was used [20].

### Expression of recombinant VvTPS proteins, *in vitro *enzyme assays and product identifications by GCMS

For TPS protein expression, Cip41 Rare *E. coli *cells [75] containing recombinant *VvTPS *plasmids were grown until 0.8 OD, induced with 0.5 mM IPTG and then grown for an additional 16 h at 16 °C. Recombinant VvTPS were partially purified using His SpinTrap columns (GE Biosciences, http://www.apbiotech.com). Protein expression was verified using silver stained SDS-PAGE gels and western blot analysis using Murine anti-polyHistidine Monoclonal antibody (1/4000 dilution, Sigma-Aldrich, http://www.sigmaaldrich.com) and 5-bromo-4-chloro-3-indolyl phosphate/nitro blue tetrazolium (CalBiochem, http://www.emdchemicals.com). For Strep-tag II clones, the pASK-IBA3plus vector combined with Cip41 Rare cells were used for expression and protein was isolated as per the manufacturer's instructions using a Strep-Tactin affinity purification column (IBA).

For functional enzyme characterization, approximately 100 μg total protein of semi-purified recombinant VvTPS were added to a single vial assay in 50 μL total volume as described [32,75,76]. Each recombinant VvTPS was incubated with GPP (109 μM), (*E*,*E*)-FPP (92 μM) or (*E*,*E*,*E*)-GGPP (20 μM) for 2 h at 30 °C. Buffers used for sesquiterpene and diterpene synthase assays contained 50 mM HEPES pH 7.0, 10 mM MgCl_2_, 20 μM MnCl_2_, 10% glycerol (v/v), and 1 mM DTT. For monoterpene synthase assays, the buffer consisted of 50 mM HEPES pH 7.0, 10 mM MgCl_2_, 20 μM MnCl_2 _100 mM KCl, 10% glycerol (v/v), and 1 mM DTT.

Gas chromatography coupled with mass spectrometry (GCMS) analysis was employed to determine the product profiles of each TPS as previously described [32,56]. Columns used to separate product mixtures included HP-5MS (Agilent), DB-Wax (Agilent), SolGelWax (SGE), and Cyclodex-B (Aglient) for chiral analyses. For sesquiterpene analysis, a cool-on column inlet (starting temperature of 35 °C and track oven program after injection) was used to prevent thermo-rearrangements of terpenes in the injector. Identities of individual terpenes were made using a combination of authentic standards and/or retention indices along with MS library matches (including WileyNist 2005, [77], and/or MassFinder4 [massfinder.com/wiki/MassFinder_4]).

### *In vivo *enzyme assays using metabolically engineered *E. coli*

A subset of *VvTPS *encoding sesquiterpene synthases were codon-optimized for expression in *E. coli*, using gene synthesis by DNA2.0 https://www.dna20.com/ and subcloned into the pET-3a or pET-Duet1 expression plasmids (Novagen, http://www.emdchemicals.com). Enzymatic activity was assessed in *E. coli *cells co-expressing the *TPS *together with the enzymes of a five-step biosynthetic pathway converting mevalonic acid to FPP. The FPP synthase gene was amplified from *S. cerevisiae *genomic DNA and ligated into the first multiple cloning site (MCS) of the pACYCDuet-1 expression plasmid (Novagen, http://www.emdchemicals.com) providing the plasmid FPPs-pACYCDuet. An operon encoding the genes for a mevalonate kinase (*MvaK1*), a phosphomevalonate kinase (*MvaK2*), a mevalonate diphosphate decarboxylase (*MvaD*) and an isopentenyl diphospahte isomerase (*idi*) was amplified from genomic DNA of *Streptococcus pneumoniae *(ATCC BAA-334) and ligated into the second MCS of the FPPs-pACYCDuet plasmid providing the plasmid pACYCDuet-4506. BL21 Star™(DE3) *E. coli *cells http://www.invitrogen.com were co-transformed with the plasmids, pACYCDuet-4506 and either of the pET series plasmids harboring candidate VvTPS coding sequences. Single colonies were used to inoculate 5 mL of LB medium supplemented with carbenicillin (50 mg/mL) and chloramphenicol (34 mg/mL). Cultures were incubated overnight at 37°C. The next day 250 mL to 1 L of Terrific Broth medium supplemented with the appropriate antibiotics were inoculated with 1/100 volume of the overnight culture. After 6 h incubation at 37°C, cultures were cooled down to 28°C and then 1 mM IPTG, 2 mg/mL mevalonate prepared by dissolving mevalonolactone http://www.sigmaaldrich.com in 0.5N NaOH at a concentration of 1 g/mL and incubating the solution for 30 min at 37°C) and 0.1 volume of decane were added to the cultures. After 48 h incubation, the decane fraction was directly analysed by GCMS on a Hewlett Packard 6890 series GC system equipped with a DB1 column 30 m × 0.25 mm × 0.25 mm film thickness http://www.agilent.com and coupled with a 5975 series mass spectrometer. The carrier gas was helium at a constant flow of 1 ml.min^-1^. Injection was in splitless mode with the injector temperature set at 120°C and the oven temperature was programmed from 60°C to 265°C at 5°C.min^-1^. Identification of VvTPS products was based on retention time index, mass spectra of authentic standards, and on published [78] or Firmenich MS database. Co-injections were also used for some of the compounds (caryophyllene, humulene, cubebol, cubebene, intermedeol). For compounds that could not be identified by GCMS, the products were purified and their structures determined by NMR spectroscopy. Sesquithujene was isolated using manual, preparative-GC. The crude sample (bi-phasic bacteria culture) was first distilled using a Fisher column to remove the decane (temperature: 95 °C; pressure: 25 mbar). The compounds were then separated using a GC equipped with a.1.83 m × 2.1 mm i.d., 10% OV -1 packed column at a flow rate of 10 mL/min. The oven temperature was programmed from 160 °C (held 10 minutes) to 230 °C at 10 °C/min. Helium was used as the carrier gas. Hyemalol was purified by silica gel flash column chromatography (Silicagel 60, 12*150 mm, 40-63 μM, Merck) using a 98:2 mixture of toluene and diethyl ether as solvent system. NMR data were acquired at 298 K using a Bruker Avance 500 MHz spectrometer. The structure was established by 1 D ^1^H- and ^13^C- NMR, as well as 2 D HSQC, COSY and HMBC experiments.

## Abbreviations

VvTPS: *Vitis vinifera *terpene synthase; AtTPS: *Arabidopsis thaliana *terpene synthase; FLcDNA: full length cDNA; PN: Pinot noir; CS: Cabernet Sauvignon; Gw: Gewürztraminer; FPP: farnesyl diphosphate; GPP: geranyl diphosphate; GGPP: geranylgeranyl diphosphate.

## Authors' contributions

DMM, OT, MBS, LD, and SA, performed experiments and analyzed data. DMM, JB, LD, MS and SA conceived of the study, interpreted results and wrote the paper. OT, LD, MS and STL reviewed the paper prior to submission and provided valuable comments on the interpretation and presentation of results. JB and STL secured funding. All authors read and approved the final manuscript.

## Supplementary Material

Additional file 1 **The Excel file contains all the information relative to the 152 TPS loci detected and curated in the 12-fold PN40024 grapevine genome**. The content of each column is: - Name: VvTPS nomenclature for the 69 complete terpene synthases. - Gene ID (12x): The official ID of the gene automatically annotated by IGGP (International Grape Genome Program) and used by GenBank/EMBL. The genes called 'newX' correspond to TPS loci completely missed by the automatic annotation pipeline. The curated gene structures and sequences are available in the FLAGdb^++ ^database http://urgv.evry.inra.fr/FLAGdb. - merged ID: If the re-annotated TPS genes fit or overlap with several IGGP consecutive genes (erroneous splitting of the automatic annotation), their IDs are mentioned here. - Prot size: The size (in amino acids) of the re-annotated TPS proteins. For not complete or coherent CDS (partial or pseudo-TPS), the size fits with the longest rebuilt protein sequence (in italic). - Chr: The chromosome number. '10R' means that the gene is on the chromosome 10 but on a not mapped scaffold. 'R' means that the gene is inside an unmapped scaffold. - Exons: Number of annotated exons in the CDS (after curation). - Subfamily: Classification of TPS (TPS-a to TPS-g) according to the phylogeny and functional studies. The table is colored according to this column. - DDxxD: 'yes' means that the exact motif is present at the expected position (end of the exon 4). '?' means that the corresponding part of the gene is absent (partial gene or pseudogene). - RRx(8)W: 'yes' means that the exact motif is present at the expected position (end of the exon 1). '?' means that the corresponding part of the gene is absent (partial gene or pseudogene). - NSE/DTE: sequence of the NSE/DTE motif present in the C-terminal region of the proteins from TPS-a, -b and -g subfamilies. - Start: first position of the curated CDS in the 12-fold pseudo-chromosomes. - Stop: last position of the curated CDS in the 12-fold pseudo-chromosomes. - Strand: 'm' means minus strand and 'p' means plus strand relatively to the pseudo-chromosomes. - Manual re-annotation result: information about the evaluation and curation process. Protein IDs are listed for the *VvTPS *genes where automatic annotation predicted a correct structure. - Type: 'full' means that the TPS gene (CDS) is complete, without sequence problem. 'full ?' means that CDS is complete excepted one punctual sequence problem. A sequencing error is therefore possible and the gene could be functional. 'partial' means that the gene is disrupted by an un-sequenced region (gap of N) and additional sequencing is necessary to have a full CDS. 'pseudo' means that the gene structure is disturbed by stop(s) in frame, frameshift(s) and/or deletion(s). As it is, the gene cannot be functional and is qualified as pseudogene. - EST: The number of available cognate transcript sequences. (+) means that the CDS is fully covered by the EST contig. - Note: Additional information about the partial genes and pseudogenes (nature of the problem, gap positions...). - Cluster: TPS with the same letter are organized in tandem in the same physical cluster. - Subcell. loc.: The result of the ChloroP prediction tool. A score greater than 0.5 means that the TPS protein is probably targeted to the plastids. 'x' means that the prediction has not been done because the N-terminal region of the protein is lacking. - Best hit in SwissProt: Biochemical function of the first hit obtained in the SwissProt database with BLASTP. Click here for file
